# Accurate Estimates of Microarray Target Concentration from a Simple Sequence-Independent Langmuir Model

**DOI:** 10.1371/journal.pone.0014464

**Published:** 2010-12-30

**Authors:** Raad Z. Gharaibeh, Anthony A. Fodor, Cynthia J. Gibas

**Affiliations:** Department of Bioinformatics and Genomics, The University of North Carolina at Charlotte, Charlotte, North Carolina, United States of America; University of Minnesota, United States of America

## Abstract

**Background:**

Microarray technology is a commonly used tool for assessing global gene expression. Many models for estimation of target concentration based on observed microarray signal have been proposed, but, in general, these models have been complex and platform-dependent.

**Principal Findings:**

We introduce a universal Langmuir model for estimation of absolute target concentration from microarray experiments. We find that this sequence-independent model, characterized by only three free parameters, yields excellent predictions for four microarray platforms, including Affymetrix, Agilent, Illumina and a custom-printed microarray. The model also accurately predicts concentration for the MAQC data sets. This approach significantly reduces the computational complexity of quantitative target concentration estimates.

**Conclusions:**

Using a simple form of the Langmuir isotherm model, with a minimum of parameters and assumptions, and without explicit modeling of individual probe properties, we were able to recover absolute transcript concentrations with high *R^2^* on four different array platforms. The results obtained here suggest that with a “spiked-in” concentration series targeting as few as 5–10 genes, reliable estimation of target concentration can be achieved for the entire microarray.

## Introduction

DNA microarrays [1] are a primary research tool for assessing global gene expression. Structurally, a microarray is a solid surface on which nucleic acid strands (*probes*) are attached. Functionally, they operate on the principle of nucleic acid complementarity between the attached probes and the components of the *target* mixture (a mixture of labeled nucleic acids). The result is formation of a stable duplex, from which a signal is detected at each probe only if there is a complementary molecule present in the labeled target mixture. This signal is then used in further analysis and inference steps.

Models that attempt to estimate target concentrations on microarrays can be, generally, divided into two main categories: The first includes models that rely on the Langmuir isotherm [2], [3], [4], [5]. The Langmuir equation describes the equilibrium between a solute and a functionalized surface. In the microarray context, it is generally formulated as a hyperbolic function:
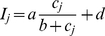
(1)where *I* is the signal intensity from a given microarray probe at target concentration *c*, and *a*, *b* and *d* are the model fitting parameters. The fitting parameter *a* is the saturation intensity (assuming *d*  =  0), *b* is the target concentration that saturates half of the probes, and *d* is the background component [2]. Some of the models in this category predict these parameters from probe sequence composition [2] or probe/target and target/target binding energy [3], [5]. Other models [4] fit the data to the Langmuir isotherm and obtain *a*, *b* and *d* for each probe using a non-linear minimization approach. In all of these models, each probe is characterized by its own *a*, *b* and *d*. If a microarray has *n* probes or probesets, then there are 3*n* parameters. Once the three parameters are determined, target concentration is predicted by inverting the isotherm.

A second category of models depends on competitive hybridization chemistry [6], [7], [8] to predict probe signal intensity, which is translated either to expression level or absolute target concentration. Those models are based on the thermodynamics of hybridization, and parameterized based on in-solution DNA hybridization behavior [9], [10]. They rely on individual probe properties and consequently are prone to over-parameterization.

We have developed a simple probe-property-independent model, the *global average model* (GLAM), which we have used to predict absolute target concentration on different microarray platforms, including Affymetrix, Agilent, Illumina and a locally developed custom microarray. In the GLAM model, the three parameters of the Langmuir isotherm are fit to all of the data from each microarray. Instead of characterizing each probe or probeset with its own *a*, *b* and *d*, we characterize a group of experiments with one *a*, *b* and *d*, thus reducing the number of free parameters to three for each microarray. The GLAM model has the advantage that, unlike other models [4], [6], [11], it can be fit with spike-in dose-response data from a small number of genes and subsequently can be used to make predictions for the entire microarray. Unlike most other currently available models [7], [12], [13], GLAM is applicable to microarrays that don't have multiple probes per probeset, and as a result, we are able to test its performance across multiple array platforms. Our predictions of target concentration on these microarray platforms equal or outperform those made using models that rely on individual free parameters for each probe.

### Analysis

We tested the performance of GLAM on control datasets from each of the most popular microarray platforms, as well as on the Microarray Quality Control (MAQC) data sets.

### The Langmuir isotherm

The Langmuir isotherm is a hyperbolic response function (Eq. 1) where *I_j_* is the signal intensity from the probes at target concentration *j*. *a*, *b* and *d* are the model fitting parameters, and *c* is the target *j^th^* concentration in pM. This model has three free parameters (*a*, *b* and *d*) fitted to different concentrations, depending on the dataset used. The fitting parameter *a* is the saturation intensity (if there is no cross-hybridization, i.e. *d*  =  0), *b* is the target concentration that saturates half of the probes, and *d* is the background component [2]. The model was fitted using the *nls* function of R [14]. In contrast with commonly used approaches, the three parameters were obtained by fitting the model to data from a number of probes (*training probes*) and not specifically to individual probes.

### Estimation of target concentration

To estimate target concentration (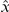), we used the approach described by Burden *et al.* [15] with a slight modification:
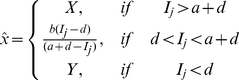
(2)where *a*, *b* and *d* are the fitted parameters of equation 1 above. *X* is an arbitrarily chosen large concentration, assigned when the probe has signal intensity above the Langmuir saturation limit. *Y* is an arbitrarily chosen small concentration, assigned when the probe has signal intensity below the predicted background limit. *X* and *Y* were set above the largest target concentration and below the smallest target concentration in each dataset, respectively.

**Figure 1:**
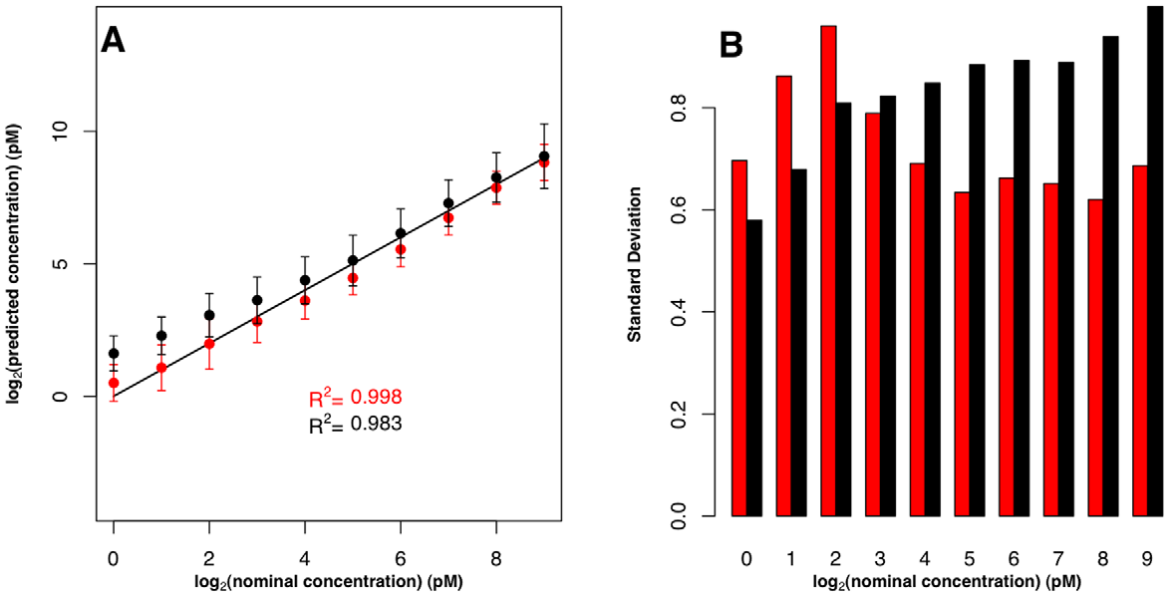
Estimation of transcript concentrations on the Affymetrix platform: Comparison to hybridization model-based approach. Estimations of 19 transcripts chosen by Li *et al.* (**A**) Results obtained from a training set of three probesets for GLAM (red) and those obtained from Li *et al.* approach (black). Error bars are standard deviations. The solid line is the identity line (*x* = *y*). (**B**) Comparison of error bar lengths for each concentration for our approach (red) and the Li *et al.* approach (black).

**Figure 2:**
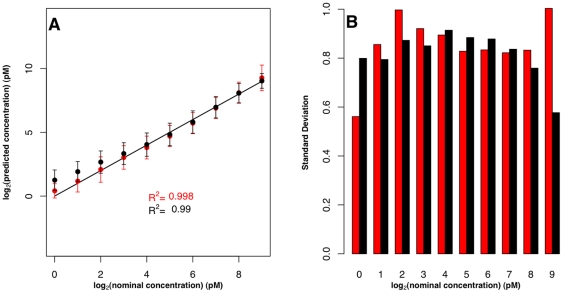
Estimation of transcript concentrations on the Affymetrix platform: Comparison to probe-property-dependent Langmuir fitting approach. (**A**) Results obtained from a training set of three probesets for GLAM (red) and those obtained using the Abdueva *et al.* approach (black). Error bars are standard deviations. The solid line is the identity line (*x* = *y*). (**B**) Comparison of error bar lengths for each concentration for our approach (red) and the Abdueva *et al.* approach (black).

**Figure 3:**
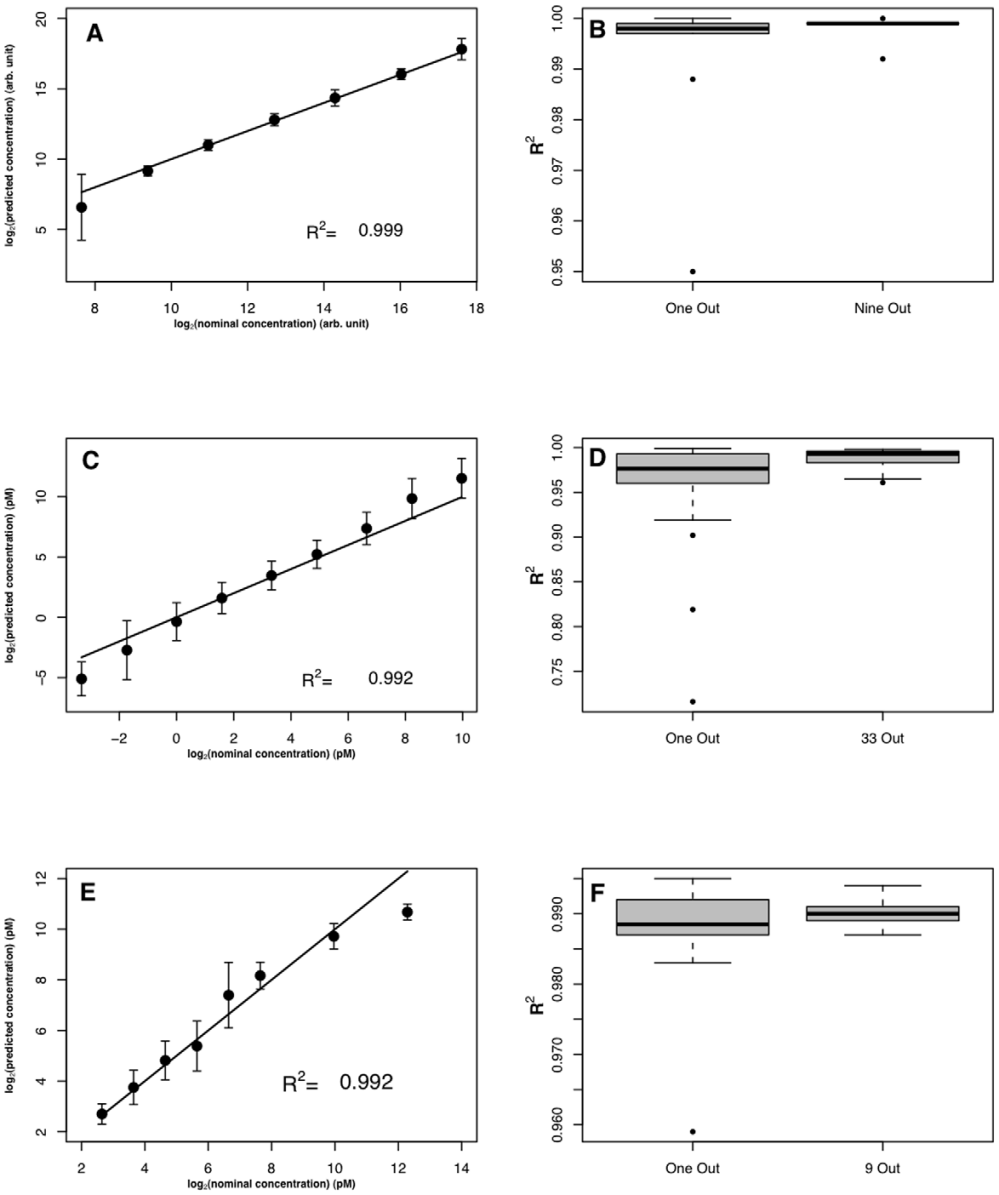
Estimation of transcript concentrations on the Agilent platform (A and B), Illumina platform (C and D) and pin-spotted platform (E and F). The first column shows results obtained from a comprehensive leave-one-out procedure. Error bars are the standard deviations of the ten transcripts. The solid line is the identity line (*x* = *y*). The second column shows box plots of *R^2^* for the ten (Agilent), thirty three (Illumina) and ten (pin-spotted) estimations of leave-one-out procedure and *R^2^* for five estimations of leave-nine-out (Agilent), leave-33-out (Illumina) and leave-nine-out (pin-spotted) procedures.

In this report we divide spike-ins into three categories: low, medium and high, following McCall *et al.* [16]. These concentration ranges are meaningful in the context of the experiment. The medium range corresponds to the linear range of the experiment. The “low” concentration range refers to the range where signal is indistinguishable from background, and the “high” concentration range includes concentrations that are outside the linear range of the experiment at the high end, where saturation occurs. In Figures 1, 2, and 3, we do not estimate target concentrations for spike-ins in the low concentration category. These data are included for comparison in Figure S3, where it can be seen that all the models interpret the values as corresponding to zero concentration.

### Datasets

Five datasets were used to evaluate the performance of the GLAM model. The first three datasets (the Affymetrix HGU133A GeneChip Latin Square dataset, the Agilent 4x44K Whole Human Genome Oligo Microarray control dataset, and Illumina's Human-6 v2 Beadchip control dataset) were generated for the External RNA Control Consortium [17]. Full descriptions of those datasets, along with the raw data, can be found here [16] and in references therein. The fourth dataset is an ArrayIt 50-mer control dataset spotted on a standard epoxysilane-coated glass slide substrate, described previously in [18]. The final dataset used to test the model is the MAQC dataset. In this dataset, four samples of universal human reference RNA (Sample A), human brain total RNA (Sample B), 3∶1 mixture of A and B (sample C) and 1∶3 mixture of A and B (sample D) were hybridized to different microarray platforms (Applied Biosystems, Affymetrix, Agilent, Eppendorf, GE Health care, Illumina and NCI Operon) and also validated using three alternative gene expression quantitation approaches (TaqMan Assays, QuantiGene Assays and StaRT-PCR Assays) [19]. For each sample, we used the raw intensities and averaged over technical replicates and test sites. The quantifications of StaRT-PCR (Standardized Reverse Transcriptase PCR) [20], in terms of number of molecules of each gene present in each sample, were used as the gold standard to which the predictions of GLAM were compared. There are 205 genes quantified in the MAQC assay, from which we used 86 genes, chosen because they are present on each of the Affymetrix, Agilent one-color and Illumina platforms. Genes interrogated by probes used for training GLAM were omitted from the comparisons shown in Table 1.

**Table 1:** Summary of GLAM predictions on the MAQC datasets.

Sample	*y*	*x*	*R^2^*	*Slope*
A	ILM	GEX	0.9	0.94
	AG1	GEX	0.94	1.08
	AFX	GEX	0.95	0.98
B	ILM	GEX	0.87	0.84
	AG1	GEX	0.88	1.05
	AFX	GEX	0.92	0.92
C	ILM	GEX	0.9	0.94
	AG1	GEX	0.92	1.05
	AFX	GEX	0.94	0.95
D	ILM	GEX	0.9	0.89
	AG1	GEX	0.91	1.07
	AFX	GEX	0.94	0.93
ALL	ILM	GEX	0.9	0.91
	AG1	GEX	0.91	1.07
	AFX	GEX	0.94	0.95
A	ILM	AFX	0.94	0.96
	AG1	AFX	0.93	1.07
	AG1	ILM	0.91	1.08
B	ILM	AFX	0.9	0.9
	AG1	AFX	0.88	1.1
	AG1	ILM	0.88	1.16
C	ILM	AFX	0.95	0.99
	AG1	AFX	0.91	1.07
	AG1	ILM	0.92	1.06
D	ILM	AFX	0.94	0.95
	AG1	AFX	0.92	1.12
	AG1	ILM	0.91	1.14
ALL	ILM	AFX	0.93	0.95
	AG1	AFX	0.91	1.09
	AG1	ILM	0.9	1.1

Results are presented in terms of *R^2^* and slope for fitting the model *y = mx*, where *y* and *x* are indicated on the header of the table. ILM: GLAM predictions for Illumina, AG1: GLAM predictions for Agilent one-color, AFX: GLAM predictions for Affymetrix and GEX: StaRT-PCR quantifications.

### Estimation of target concentration on the Affymetrix platform

The Affymetrix U133A Latin square control dataset has 42 transcripts spiked in at concentration range of 0.125–512 pM in a Latin square design [16], [21]. We apply the GLAM model presented in equation 1 to this dataset. We obtained *a*, *b* and *d* by fitting the model to a training set composed of three randomly chosen probesets (Fig. 1A and 2A; red symbols). Figures 1 and 2 show that GLAM is able to recover absolute target concentrations with *R^2^* of 0.99.

### Comparison of GLAM performance to established models

To evaluate the consequences of ignoring probe specific effects we compared the performance of GLAM on the Affymetrix U133 dataset to that of other models. The two models chosen are the top-performing models in each of the categories described in the introduction. The Li *et al.* model is the best-performing previously published model that depends on modeling competitive hybridization chemistry, while the Abdueva *et al.* model is the best-performing previously published model that uses a Langmuir isotherm based approach.

In Figure 1A we compare GLAM target concentration estimates to estimates from Li *et al.* [6]. Their approach depends on competitive hybridization chemistry, and target concentration is determined by the following equation [6]:
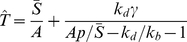
(3)where 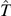 is the predicted target concentration, 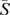 is the observed signal intensity after scanner bias and background subtraction, *A* is the detection coefficient of fluorescence, *k_d_* is the probe affinity coefficient, 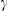 is a cross-hybridization factor, *p* is the total number of probes in molar concentration units and *k_b_* is the binding rate constant for target molecules [6].

The estimates generated by Li *et al.* in [6] are based on a subset of 19 transcripts, which were selected based on target sequence alignment matching and probe signal intensity, and sorted based on probe thermodynamic properties. We estimated transcript concentrations for these 19 transcripts using GLAM, choosing three randomly selected probesets as a training set (Fig. 1).

Our results show that both approaches are able to recover target concentration with high *R^2^* (0.998 for GLAM and 0.983 for Li *et al.* [6]). Absolute target concentrations obtained using our approach have a slope of 0.958, and those obtained using the approach of Li *et al.* [6] have a slope of 1.045. The slope value describes the accuracy of the predictions [21]; a value of 1 is considered to be the perfect score. Values below or above 1 indicate underestimation or overestimation, respectively. Although the Li *et al.* model attempts to control for factors that might increase error (such as scanner bias), the errors between the two approaches are comparable (Fig. 1B) with GLAM having slightly higher errors for target concentrations less than 4 pM, but much lower errors for target concentrations >4 pM.

Abdueva *et al.*, [4] developed a Langmuir isotherm model based approach similar to GLAM. The main difference between the two is in the way they treat individual probe effects. In the Abdueva *et al.* [4] approach, *a*, *b* and *d* are estimated for each probe, and the final transcript concentration is calibrated based on *log* predicted saturation intensity and *log* non-specific intensity of the probe. Those two values are predicted from probe thermodynamic properties, based on sequence content. In GLAM, *a*, *b* and *d* are global, based only on a training set. The training set is chosen from within the same experimental context but does not overlap the test set for which predictions are being made. We applied both approaches to the U133A Affymetrix control dataset, and estimated the absolute concentrations of the 42 transcripts. The results are presented in Figure 2. Both approaches perform well (Fig. 2A) but GLAM has a slightly higher *R^2^* (0.998) than the Abdueva *et al.* approach (0.990) despite using significantly fewer free parameters. Examining the slope of the predicted target concentrations shows that GLAM predictions have a slope of 0.997, while the Abdueva *et al.* [4] predictions' slope is 1.007. Both approaches have similar error values, as shown in Figure 2B.

The Abdueva *et al.* [4] predictions are based on normalized signal intensities, while ours are based on raw signal intensities. To explore how normalization would affect the performance of the two models, we predicted target concentrations using GLAM, but beginning with the quantile normalized signal intensities used by Abdueva *et al.* [4] (Figure S1). Similar results are obtained, with slight differences in the length of error bars and a slope of 0.994 for GLAM. The use of normalization does not appear to affect results significantly.

We also compared the performance of GLAM to the model of Abdueva *et al.* [4], but applied the Abdueva model without calibrating the final transcript concentration using the data transformation based on probe thermodynamic properties. When the calibration step in the Abdueva *et al.* approach is not used, the only difference between the two approaches is that GLAM has a single set of parameters for *a*, *b* and *d* while Abdueva *et al.* model each probe individually. Removing the thermodynamics-based calibration model of probe properties causes the performance of the Abdueva *et al.* model [4] to degrade; its *R^2^* dropped to 0.843 and slope dropped to 0.53. GLAM was, by its nature, unaffected by lack of probe-specific data (Figure S2).

While our method yielded excellent predictions of absolute transcript concentration, we did not predict concentration for all transcripts in the low concentration category (Fig. 1 and 2). This is because there is a poor correlation between signal intensity and target concentration at the low end [16], and because the signal obtained from these targets can't be differentiated from background noise in the low concentration milieu [22]. Also, microarray scanner nonlinearity is at its worst at low intensity [6], [23]. For comparison, we show the results of predicting the full range of concentrations in Figure S3. All three models examined show a decrease in terms of *R^2^* and slope values when low concentration transcripts are considered, and all three models have the same difficulty predicting low target concentrations.

### Alternate model implementation and data manipulation

The source code and data for the Abdueva *et al.* [4] and Li *et al.* [6] models were obtained from the authors. Signal intensities were normalized using quantile normalization [24] for the Abdueva *et al.* procedure and all the 42 probesets were used (including Affymetrix control probesets). For Li *et al.*, signal intensities were used without normalization and prepared according to the author's instructions [6]. Briefly, the raw signal intensities from 355 probes corresponding to 19 transcripts fitted the authors filtering procedure and were used for estimating target concentration. Probe intensity was taken as the average across technical replicates. For our model, all signal intensities were used without normalization (unless indicated). The signal intensity of each probe was taken as the average signal across technical replicates.

The MAQC datasets were obtained from the MAQC website (http://edkb.fda.gov/MAQC/MainStudy/upload/). We used the raw signal intensities and averaged over technical replicates and test sites. *R^2^* and slope values presented here were calculated using the *lm* function of R [14] using the default settings, except that the intercept term was omitted, following Irizarry *et al.* [25]and others [5].

### Estimation of target concentration on the Agilent platform

A key difference between GLAM and the Abdueva *et al.* [4] and Li *et al.* [6] models is that, due to its simplicity, GLAM can straightforwardly be applied to data types other than Affymetrix data without special modifications to the model. We tested the applicability of GLAM to the Agilent platform, which has different probe and surface properties than the Affymetrix platform. A publically available Agilent control dataset is composed of ten transcripts spiked in at ten concentrations [16]. We predicted transcript concentrations using GLAM, again without taking individual probe effects into consideration. Figure 3A shows the results, using a summary of leave-one-out procedures, where every nine probes in turn were used as a training set and the resulting *a*, *b* and *d* were used to estimate the concentration of the remaining tenth transcript. The average estimated concentrations agree well with the reported nominal concentrations with an *R^2^* of 0.999 and a slope of 0.997.

This result was obtained by training GLAM on nine probes and predicting the remaining tenth transcript, which raised the possibility of overfitting. We then tested the effect of changing the fraction of total available data that we included in the training set, since a spike-in control procedure would be most useful if it could be trained on a small fraction of the array data. Figure 3B shows box plots of *R^2^* for the ten estimations of the leave-one-out procedure described above, and *R^2^* for five estimations of a leave-nine-out procedure. In the leave-nine-out procedure, *a*, *b* and *d* are estimated from a training set of one probe and used to predict the concentrations of the remaining nine transcripts. The leave-nine-out procedure uses a small training set that is highly sensitive to the choice of probe for training. Probes showing non-Langmuir-like behavior can be avoided without explicit modeling and knowledge of their sequence, so five probes returning negative values for either *a*, *b* and *d* were not included in the training set. Transcript concentration estimation with parameters obtained from well-behaved single probes show excellent *R^2^* with a minimum of 0.992, depending on which probe was used for parameter estimation.

### Estimation of target concentration on the Illumina platform

We next tested GLAM on an Illumina control dataset composed of 34 transcripts spiked at 11 different concentrations [16]. We follow the same procedures as with the Agilent platform, and the results are shown in Figure 3C–D. Application of a comprehensive leave-one-out procedure (Fig. 3C) shows that our approach to estimating transcript concentration performs well on the Illumina platform; the average estimated concentrations show an *R^2^* of 0.992 and a slope of 1.165. The *R^2^* values obtained from 34 trials of the comprehensive leave-one-out procedure are shown in Figure 3D. Out of 34 probes, 18 probes returned negative values for one of the Langmuir parameters and therefore were not used to train the model for target concentration prediction. We were able to use the remaining 16 probes in a leave-33-out procedure with excellent results, and the *R^2^* values are shown in Figure 3D.

It is clear from Figure 3C that GLAM underestimates transcript concentrations of 0.1 and 0.3 pM and overestimates transcript concentrations of 300 and 1000 pM on the Illumina platform. However, the regression slope values reported by McCall *et al.* [16] for this platform suggest that there is simply poor agreement between signal intensity and nominal spike-in concentration in those ranges, which may mean that the linear range of this platform is relatively small.

### Estimation of target concentration on a pin-spotted platform

Finally, we examined the performance of GLAM on a pin-spotted array control data set. The pin-spotted array is a custom 50 mer array that was developed in our laboratory and described in [18]. The platform is similar to many custom microarrays, where probes are contact-spotted using a robot. The platform differs from commercially available platforms in the attachment chemistry. The control experiment that uses this array has ten targets spiked at eight different concentrations. We follow the same steps used for the above datasets and we estimate target concentrations for this dataset by obtaining *a*, *b* and *d* using either a leave-one-out or leave-nine-out procedure. Figure 3E shows the averaged predicted target concentrations for a leave-one-out procedure with an *R^2^* of 0.992 and a slope of 0.969. A leave-nine-out procedure (Fig. 3F) shows that even one probe was sufficient to retain *R^2^*≥0.95. Of the ten probes, one returned unphysical values for one of the parameters and was not used for estimating target concentration.

Although the *R^2^* and slope were lowest for this dataset, the model was able to produce acceptable target estimates. We believe the slight difference in model behavior for this platform was due to the different attachment chemistry, and to the presence of competing mismatch probes for each target in this dataset.

### Consequences of choosing different GLAM training sets

**Figure 4:**
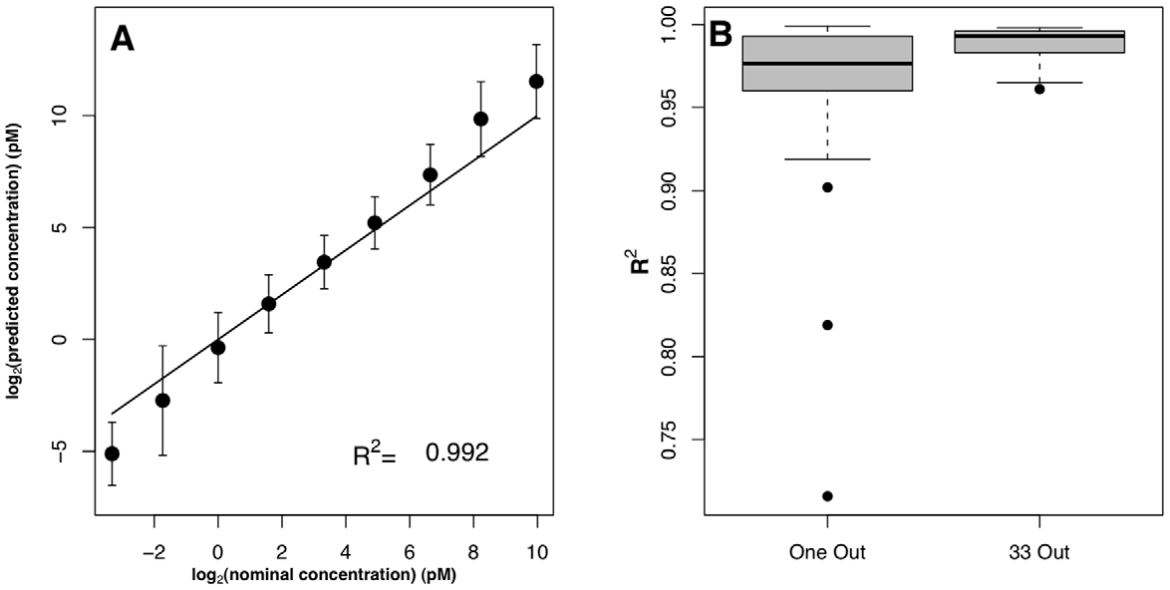
Effect of varying the number of probesets included in the training set on the performance of GLAM. Each Box plot shows the *R^2^* (*y* axis) for 42 choose K (*x* axis) of the estimated target concentrations using the Affymetrix U133A control dataset. Each box plot has 42 data points.

For the purpose of determining the values of *a*, *b* and *d*, GLAM requires a training set of known spike-ins. In this report we follow a standard *N choose K*, where *N* is the total number of spike-ins and *K* is the number of probes or probesets included in the training set. *K* has a value between one and *N* minus one. To illustrate, consider Figure 4, which shows the results on Affymetrix U133A control dataset. In this dataset there are 42 spike-ins, thus *K* (*x axis* of Fig. 4) has a range from 1 to 41. When there are more than 42 possible combinations, we choose 42 at random and we take them as a representative for all possible combinations. Therefore the box plot in Figure 4 represents all the 42 combinations of *42 choose 1*. We call this *leave-41-out*, which means that GLAM was trained on one probeset and predicted the remaining 41 spike-ins. The second box plot is for *42 choose 2*, since there are 861 different combinations, we shuffle the list of all the 42 probesets, then we choose two probesets at random and run the model, we repeat this process 42 times, thus each box plot in Figure 4 has 42 data points. The last box plot is for *42 choose 41*, we call this *leave-one-out*, which means that the GLAM was trained on 41 probesets and predicted the concentration of the remaining spike-in. We examined the effect on GLAM performance of varying the number of probes, or probesets, included in the training set. We considered all the possible numbers of training probes (or probesets). The predictive performance of GLAM under different training conditions is shown in terms of *R^2^* (Figure 4) for the Affymetrix U133A Latin square control dataset.

**Figure 5:**
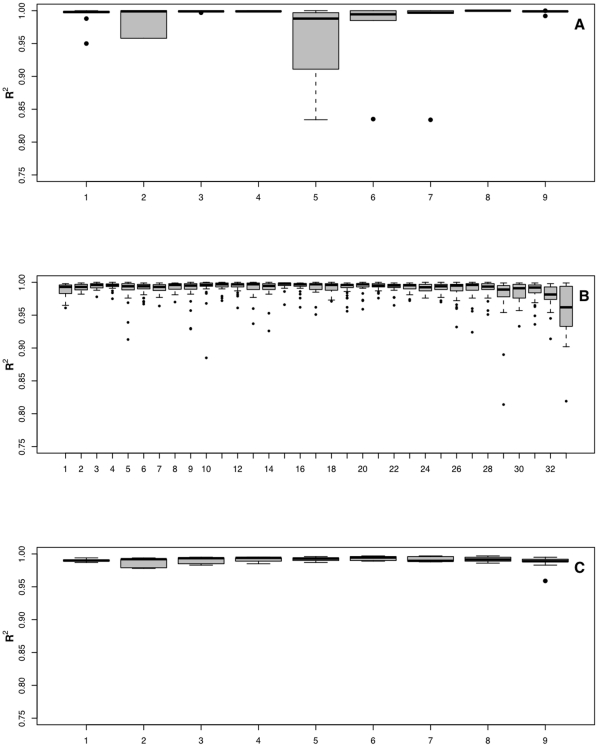
Effect of varying training set size on performance of GLAM for the (A) Agilent platform (B), Illumina platform (C) and pin-spotted platform. Each Box plot shows the *R^2^* (*y* axis) for 10 choose K (*x* axis) (**A**), 34 choose K (*x* axis) (**B**) and 10 choose K (*x* axis) (**C**) of the estimated target concentrations.

The results summarized in Figure 4 demonstrate that five probesets are enough for GLAM to return reliable results. The effect of training set size on the performance of GLAM for the other three datasets is shown in Figure 5A–C.

### Estimation of target concentration on the MAQC dataset

To demonstrate GLAM performance on a control dataset that more closely resembles a real-world microarray experiment, we predicted target concentration for the MicroArray Quality Control (MAQC) datasets [19], on Affymetrix, Agilent one-color and Illumina platforms. The MAQC datasets were collected in microarray experiments followed by extensive independent quantification of targets using a StaRT-PCR (Standardized Reverse Transcription PCR) approach. The MAQC datasets provide a group of independently quantified genes that can be used to estimate *a*, *b* and *d* for GLAM. The MAQC set is an excellent proxy problem for a real-world microarray experiment in which researchers would predict transcript concentrations based on a spike-in calibration mixture. Assuming a worst-case scenario, we used one probe per probeset (selected randomly) for the purpose of estimating GLAM parameters *a*, *b* and *d*. *X* and *Y* (for equation 3) were set to values greater than the largest number of molecules (based on StaRT-PCR quantification) of the selected gene and less than the smallest number of molecules (based on StaRT-PCR quantification) of the selected gene, respectively. The results of GLAM prediction are summarized in Table 1.

We assessed three types of comparisons. First, we compared GLAM predictions and StaRT-PCR quantifications for each sample separately, to assess the performance of the model at the sample level. We then compared the GLAM predictions for each platform at the sample level, to demonstrate the consistency of the predictions across different platforms. Finally, we constructed a global comparison between GLAM predictions and StaRT-PCR quantifications among sample groups from each of the three platforms. This is done to assess the model performance in a situation similar to a real microarray experiment where multiple samples are used (i.e. samples from different tissues or a time series experiment).

Table 1 shows these results in terms of *R^2^* and slope for the model *y = mx* for each comparison. For example, the first row of Table 1 reports *R^2^* and slope for the model *y = mx*, where *y* is the GLAM predictions for Sample A on the Illumina platform and *x* is the StaRT-PCR values for the same sample. Generally, there is good agreement between GLAM predictions and StaRT-PCR quantifications at the sample level, with 0.87≤*R^2^*≤0.95 and 0.84≤slope≤1.08. Applying the GLAM model to predict target concentrations on the Affymetrix platform gave the best overall results.

Agreement between the three different platforms was assessed by pairwise comparisons of model performance on each platform. GLAM predictions using Affymetrix, Agilent one-color and Illumina show 0.88≤*R^2^*≤0.95 and 0.9≤slope≤1.1. GLAM predictions for the Affymetrix and Illumina platforms were in closer agreement than GLAM predictions for either of these two platforms and the Agilent one-color platform (Table 1).

GLAM predictions using the Affymetrix platform had an *R^2^* of 0.94 with StaRT-PCR quantifications, Agilent one-color, *R^2^* of 0.91 and Illumina, *R^2^* of 0.9. Predictions on Affymetrix and Illumina were in good agreement with each other (*R^2^* = 0.93). Affymetrix and Agilent one-color and Illumina and Agilent one-color are still in good agreement but with *R^2^* of 0.91 and 0.9, respectively.

All the reported values for the model *y = mx* in Table 1 were statistically significant. This is shown in the last column of Table 1, where the *p*-values for testing the null hypothesis that the slope (the term *m*) is equal to zero are reported.

## Discussion

Many approaches have been used to relate microarray probe properties to hybridization signal intensity [26]. In this report, we show that a simple physical model that employs average array-wide binding parameters is comparable in performance to models that use per-probe parameters. We compared results from our GLAM approach to the results of two methods [4], [6] that have been demonstrated to be the best-performing of the Langmuir-based and hybridization chemistry-based model approaches. Our results show that, despite the differences in probe design and sequence, probe effects average out and may be modeled globally to recover specific transcript concentrations.

Obtaining *a*, *b* and *d* for a training set of probes, then using those values to predict the behavior of other probes, implies that all probes have the same *a*, *b* and *d*. We know from past studies that each probe has its own *a*, *b* and *d* and that these values are generally dependent on sequence composition [2]. So why, then, does an average Langmuir model perform as well as, or better than, sequence specific models? If we stipulate that state of the art microarray probe design procedures usually require that all probes have similar GC content, resulting in very similar hybridization profiles[27], on a well-designed array global *a*, *b* and *d* may adequately represent the individual probe properties. Many commonly-used, commercially available microarray platforms have been refined to the point that most of the probes used have similar, and close to idealized, hybridization properties [28], [29], [30]. Exceptions can generally be predicted and excluded from analysis based on our understanding of the physics of microarray hybridization [31], [32]. Fine-tuning of the parameters to reflect the differences of each probe based on its sequence composition and thermodynamic properties, or based on the observed response of each probe, may be unnecessary.

What we find to be important to the success of the GLAM model is that we estimate *a*, *b* and *d* from probes that have a Langmuir-like response to varying target concentration. As we have shown, this should be enough to ensure reliable results (Figure 4 and 5). The number of probes or probesets used in the training set does not seem to affect the performance of our model, as long as the probes included in the training set show Langmuir-like response, or if the number of training probes used is sufficient to average the effect of other probes that do not follow Langmuir-like response. Using probes that do not follow Langmuir-like behavior to estimate *a*, *b* and *d* (i.e. probes that have negative values for any of these parameters) will degrade the performance of GLAM. This can be avoided by including more probesets in the GLAM training set, or by using a set of control probes that are known to have a Langmuir-like response. In the relatively small data sets examined in this study, we were not able to identify a sequence-dependent predictor for non-Langmuir behavior. However, there are many other possible factors. Manufacturing conditions, slide chemistry or processing chemistry may not have an equal impact on all probes, and this is certainly a subject for further experiment. A recent study suggests that not all probes behave according to the Langmuir model, and our results are consistent with that observation [33]. The authors of [33] observe a higher frequency of non-Langmuir behavior, but their results are somewhat difficult to generalize or to compare with our results, as they have used a very short probe and a highly structured, very long rRNA target. They also observe very different outcomes depending on the slide chemistry. For one manufacturer Langmuir behavior was observed frequently, and for the other it was not. However, in the control and MAQC data sets examined in this study, Langmuir behavior is sufficiently widespread to provide a training set of Langmuir-conforming probes, and the model can then accurately predict the remainder of the data whether Langmuir-conforming or not.

Applying the model to the MAQC datasets showed that our model predictions are in good agreement with StaRT-PCR quantification (Table 1), with *R^2^*≥0.90. Inter-platform predictions of our model demonstrate consistency of predictions across the three different platforms used (Affymetrix, Agilent one-color and Illumina) with *R^2^*≥0.90. This is in agreement with the finding of the MAQC consortium [19].

Using a simple form of the Langmuir isotherm model, with a minimum of parameters and assumptions and without explicit modeling of individual probe properties, we were able to recover absolute transcript concentrations with high *R^2^* on four different array platforms. To our knowledge, this is the first report to produce a working model that is equally valid for four of the most frequently used microarray formats. Given the choice of models with equivalent performance, Occam's razor dictates that the model with the fewest free parameters is to be preferred. Our results therefore suggest that, despite considerable efforts by the bioinformatics community [4], [6], [26], [34], the additional complexity introduced by models that attempt to use individual probe characteristics to improve estimates of absolute concentration is not justified by a corresponding increase in performance. Given consistent concentration-dependent behavior, it should be possible to project target concentration from intensity with an accuracy equivalent to or better than sequence-specific models on any of these platforms, based on a spike-in calibration mixture containing only a few probes.

### Code and Data

Instructions for running GLAM are included as File S1. The code and data used in this study are available as an R package and can be downloaded from http://gibas-research.uncc.edu/glam/index.html, and are also included as File S2.

## Supporting Information

Figure S1 Estimation of transcript concentrations on Affymetrix platform using quantile normalized signal intensities. (A) Results obtained using a training set of three probesets with GLAM are shown in red and those obtained using the Abdueva et al. approach are shown in black. Error bars are the standard deviations of the 42 transcripts. The solid line is the identity line (*x = y*). (B) Comparison of error bar lengths for each concentration for GLAM (red) and the Abdueva et al. approach (black).
(0.01 MB EPS)

Figure S2 Performance comparison between GLAM and probe-property-dependent approach. Results were obtained using a training set of three randomly chosen probesets for GLAM. Error bars are the standard deviations of the 42 transcripts. The dashed line is the identity line (*x = y*); solid lines are the regression lines. *R^2^* and slope values are colored coded according to the schema above and indicated on the graph.
(0.01 MB EPS)

Figure S3 Estimation of transcript concentrations on Affymetrix platform using the full range of concentrations (14 total concentrations). Results were obtained using a training set of three randomly chosen probesets. Results for GLAM are shown as red circles. Abdueva et al. approach results are shown as black squares and Li et al. approach results are shown as blue triangles. Error bars are the standard deviations of the 42 transcripts in the case of GLAM and Abdueva et al. approach and 19 transcripts in the case of Li et al. approach. The solid line is the identity line (*x = y*). *R^2^* and slope values are colored coded according to the schema above and indicated on the graph.
(0.01 MB EPS)

File S1 GLAM procedure outline. This file describes the procedure for constructing GLAM input and applying GLAM to the Affymetrix U133A data set.
(0.03 MB DOC)

File S2 GLAM code and data archive.
(0.04 MB GZ)
