# Supplementary material for: Accurate Estimates of Microarray Target Concentration from a Simple Sequence-Independent Langmuir Model
Source: PLoS One. 2010 Dec 30;5(12):e14464. doi: 10.1371/journal.pone.0014464 (PMC3012684; doi:10.1371/journal.pone.0014464)
Supplement: File S1 — GLAM procedure outline. This file describes the procedure for constructing GLAM input and applying GLAM to the Affymetrix U133A data set. (0.03 MB DOC) [file pone.0014464.s004.doc]

GLAM Procedure

This document illustrates the major steps in using GLAM to estimate target concentrations from a microarray experiments. We will use the Affymetrix U133A Latin square control dataset as an example. This procedure is equivalent to running the function glamAffy() in the code provided along with the manuscript.

Suppose you have 14 microarrays representing 14 experimental conditions. Lets assume each microarray contains *n* probes, and three spike-ins.

1. Arrange the intensities of all the 14 arrays and have them in a matrix, data frame or a table of *n* x 14, where the rows represent the expression of each probe and the columns represent the experimental conditions.
2. Arrange the intensities of the spike-in in a similar data structure, which will be 3x14 in the example presented here. In addition, you will need the spike-in concentrations in a vector. Each value in the concentration vector corresponds to probe intensity in the spike-in matrix. In this example the concentration vector is composed of 14 values.
3. Estimate the parameters *a*, *b* and *c* from the spike-in matrix using the function calPara(intsMat, cons), where intsMat is the intensity matrix of the spike-ins and cons is the vector of spike-in concentrations.
4. Using the estimated *a*, *b* and *c* parameters, calculate the concentration of each probe in the probe matrix using the function calConsForPick(intsMat, cons, a, b , c), where intsMat is the intensity matrix of the probes and cons is the vector of spike-in concentrations (this is used to calculate the correlation between the predicted and the actual concentrations), *a*, *b* and *c* are the estimated parameters from step 3 above.

The following R code will execute the steps above using the glam package:

library(glam)

data(LS133) #load the LS133A data

concs <- c(0, 0.125, 0.25, 0.5, 1, 2, 4, 8, 16, 32, 64, 128, 256, 512) # define the concentration matrix

pick3 <- c("AFFX-r2-TagH_at", "204430_s_at", "204959_at") #those are the three spike-ins

whosInIdx <- getInIndx(pick3, LS133Ints) # prepare the training matrix indices

spikeIns.ints <- as.matrix(LS133Ints[whosInIdx, 3:16]) # this is the spike-in intensity matrix in step 2 above

para <- calPara(spikeIns.ints, concs) #estimate a, b and c parameters

whosOutIdx <- getOutIndx(pick3, LS133Ints) # prepare the probe matrix indices

probe.ints <- as.matrix(LS133Ints[whosOutIdx, 3:16]) # this is the actual probe intensity matrix in step 1 above

calConsForPick(probe.ints, concs, para[1], para[2], para[3]) #predicting each probe concentration, this is step 4 above

This same simple procedure can be applied to any data set, you just need to prepare your spike-in intensities matrix, spike-in concentrations vector and your probe intensities matrix, as outlined above.
